# PKC–NF-κB are involved in CCL2-induced Na_v_1.8 expression and channel function in dorsal root ganglion neurons

**DOI:** 10.1042/BSR20140005

**Published:** 2014-06-18

**Authors:** Rui Zhao, Guo-Xian Pei, Rui Cong, Hang Zhang, Cheng-Wu Zang, Tong Tian

**Affiliations:** *Department of Orthopedics, Xijing Hospital, Fourth Military Medical University, Xi’an, China

**Keywords:** CCL2, CCR2, dorsal root ganglion (DRG), Na_v_1.8, nociception, PKC, CCL2, chemokine (C–C motif) ligand 2, CCR2, chemokine (C–C motif) receptor 2, DRG, dorsal root ganglion, GAPDH, glyceraldehyde-3-phosphate dehydrogenase, NF-κB, nuclear factor κB, PKC, protein kinase C, TEA-Cl, tetraethylammonium-Cl, TRPV1, transient receptor potential vanilloid 1, TTX-R, tetrodotoxin-resistant, VGSC, voltage-gated sodium channel

## Abstract

CCL2 [chemokine (C–C motif) ligand 2] contributes to the inflammation-induced neuropathic pain through activating VGSC (voltage-gated sodium channel)-mediated nerve impulse conduction, but the underlying mechanism is currently unknown. Our study aimed to investigate whether PKC (protein kinase C)–NF-κB (nuclear factor κB) is involved in CCL2-induced regulation of voltage-gated sodium Nav1.8 currents and expression. DRG (dorsal root ganglion) neurons were prepared from adult male Sprague–Dawley rats and incubated with various concentration of CCL2 for 24 h. Whole-cell patch-clamps were performed to record the Nav1.8 currents in response to the induction by CCL2. After being pretreated with 5 and10 nM CCL2 for 16 h, CCR2 [chemokine (C–C motif) receptor 2] and Nav1.8 expression significantly increased and the peak currents of Na_v_1.8 elevated from the baseline 46.53±4.53 pA/pF to 64.28±3.12 pA/pF following 10 nM CCL2 (*P*<0.05). Compared with the control, significant change in Na_v_1.8 current density was observed when the CCR2 inhibitor INCB3344 (10 nM) was applied. Furthermore, inhibition of PKC by AEB071 significantly eliminated CCL2-induced elevated Nav1.8 currents. *In vitro* PKC kinase assays and autoradiograms suggested that Nav1.8 within DRG neurons was a substrate of PKC and direct phosphorylation of the Na_v_1.8 channel by PKC regulates its function in these neurons. Moreover, p65 expression was significantly higher in CCL2-induced neurons (*P*<0.05), and was reversed by treatment with INCB3344 and AEB071. PKC–NF-κB are involved in CCL2-induced elevation of Na_v_1.8 current density by promoting the phosphorylation of Na_v_1.8 and its expression.

## Abstract 2

Cytokine CCL2 is responsible for promoting voltage-gated sodium Na_v_1.8 current density and expression, which mediates nerve impulse conduction and induces inflammatory nociception. PKC phosphorylates Na_v_1.8 to increase its current density and PKC–NF-κB are involved in inducing the up-regulation of Na_v_1.8.

## INTRODUCTION

Peripheral nerve injury, tissue inflammation and neuropathic disorders often result in neuropathic pain, which affects approximately 7% of the general populations and is insufficiently treated with currently available drugs [1]. The typical symptoms of neuropathic pain are spontaneous pain, allodynia and thermal hyperalgesia [2]. Chemokines and cytokines secreted by infiltrating immune cells, such as macrophages, lymphocytes and neutrophils, enhance the activity of nociceptive DRG (dorsal root ganglion neurons), eventually leading to hyperpathia of afferent neurons [3]. Among them, the chemokine CCL2 [chemokine (C–C motif) ligand]was shown to play a key role in spinal nociceptive processing, mainly by sensitizing primary afferent neurons via binding to its preferred receptor CCR2 [chemokine (C–C motif) receptor 2] to enhance the pain hypersensitivity [4,5]. Moreover, CCR2 is expressed in primary afferent DRG neurons, and the antagonists or blocking antibodies of CCR2 successfully inhibited the nociceptive signalling in peripheral neurons [6].

VGSCs (voltage-gated sodium channels) are very important for electrogenesis and nerve impulse conduction, and are suggested to be involved in the pathogenesis of chronic pain in primary sensory neurons [7]. Among them, Na_v_1.8 is a critical VGSC isoform that is preferentially expressed in primary somatosensory afferent neurons to sense stimuli, which produces a slow-inactivating, TTX-R (tetrodotoxin-resistant) current and is responsible for action potential propagation within sensory neurons of the DRGs. Compelling evidence showed that there were no change of acute nociceptive thresholds and mild deficits in inflammation-induced hypersensitivity in a Na_v_1.8 mutant mouse model [8]. Moreover, both Na_v_1.8-null and Na_v_1.8-knocked-down mice exhibited decreases in behavioural responses to noxious thermal and mechanical stimuli [9,10]. Recently, several studies reported that CCL2 contributed to the regulation of expression and current density of Na_v_1.8 in DRG neurons [11]. However, the underlying mechanisms of the regulation of CCL2 on Na_v_1.8 are still unknown.

VGSCs can be modulated by receptors coupled to intracellular signalling molecules through the activation of cytoplasmic protein kinases to phosphorylate the specific residues on the α-subunit of the VGSCs. PKA (protein kinase A) and PKC (protein kinase C) were shown to target VGSCs [12]. Over the past two decades, the functional role of PKC in pain and analgesia has been subjected to intense studies, showing that PKC activation could sensitize peripheral afferent neurons and enhance currents following noxious thermal stimulation [13]. PKCε can modulate nociception through activation of the TRPV1 (transient receptor potential vanilloid 1) receptor, a member of the transient receptor potential ion channel superfamily [14]. Additionally, PKC participates in the up-regulation of TTX-R, persistent Na^+^ currents in rat and mouse sensory neurons [15]. A recent study has ascribed the mechanical hyperalgesia to increased channel function of Na_v_1.8 by PKCε phosphorylation and identified that Na_v_1.8 acted as a substrate of PKC [16]. However, whether PKCε is involved in CCL2–CCR2-mediated regulation of Na_v_1.8 still requires more investigation.

Our study aimed to investigate the role of CCL2/CCR2 in Na_v_1.8 modulation in rat DRG, to clarify whether the regulation of CCL2 on Na_v_1.8 is dependent on PKC activation, and to make determine if the NF-κB (nuclear factor κB) pathway is involved in the regulation of Na_v_1.8 expression.

## MATERIALS AND METHODS

### Primary neuronal culture of the DRG

Adult male Sprague–Dawley rats were housed in animal care facilities under moderate temperature and humidity and changing light conditions with access to food and clean water *ad libitum*. All experiments were approved by the Institutional Animal Care and Use Committee of the Fourth Military Medical University, and in compliance with ‘Guidelines for the Care and Use of Laboratory Animals’.

DRG neurons from normal and PSL-treated rats were isolated as described previously [3]. In brief, rats were exposed to CO_2_ and decapitated. Lumbar DRGs (L4–L5) were quickly dissected and incubated in Ca^2+^/Mg^2+^-free PBS containing 1.6 M glucose. Then the DRGs were digested with collagenase A (1 mg/ml; Sigma) for 90 min and trypsin (0.25%, w/v) for 30 min at 37°C in PBS with glucose. Ganglia were then dispersed by a fire-polished Pasteur pipette in DMEM (Dulbecco's modified Eagle's medium)/F12 (1:1) medium with 10% (v/v) FBS and 1% (v/v) penicillin/streptomycin. The dissociated cells were centrifuged and plated onto poly-L-ornithine-coated and collagen-coated dishes, then incubated at 37°C in 5% (v/v) CO_2_. The cultured DRG neurons were pretreated with different concentrations of CCL2 (R&D Systems), CCR2 inhibitor INCB3344 or PKC inhibitor AEB071 and then used for electrophysiological recordings or RT–PCR and Western blotting assays.

### Western blotting

Expression of CCR2 (E68), Na_v_1.8 (S134-12) and GAPDH (glyceraldehyde-3-phosphate dehydrogenase) (6C5) were analysed by Western blotting using commercially available antibodies from Santa Cruz Biotechnology, Inc.. The cell lysates were subjected to SDS–PAGE and subsequently transferred to a PVDF membrane. Blots were visualized using Amersham Western blot detection reagent (GE Healthcare).

### RT–PCR

RT–PCR was performed to investigate the expression of CCR2 and Na_v_1.8. Total RNA of primary neurons of DRG, was extracted and purified using an RNeasy mini kit according to the instructions of the manufacturer (Qiagen). Approximately 5 μg of isolated RNA was subsequently reverse-transcribed using an dT (oligo)- primer and reverse transcriptase (Qiagen) according to the manufacturer's protocol. RT–PCR was performed with the ABI Prism 7500 sequence detection system (PE Applied Biosystems) using a SYBR Green real-time PCR Master Mix kit (Takara Biotechnology) based on the manufacturer's instructions. CCR2 primers (sense 5′-GTTGGTGAGAAGTTCCGAAGGT-3′; anti-sense 5′-GGTCTGCTGTCTCCCTATAGAA -3′); α-unit of Na_v_1.8 primer (sense 5′-CCGGTGGAAGCAGGAAGA-3′; anti-sense 5′-AGGAGCGGTGCAGCATGTA-3′) were used, with GAPDH primers (sense 5′-GCCAAAAGGGTCATCATCTC-3′; anti-sense 5′-GTAGAGGCAGGGATGATGTTC-3′) as the internal control. Amplification cycles were: 94°C for 3 min, then 33 cycles at 94°C for 1 min, 58°C for 1 min, and 72°C for 1.5 min, followed by 72°C for 15 min. Aliquots of the PCR products were electrophoresed on 1.5% (w/v) agarose gels, and PCR fragments were visualized by ethidium bromide staining. Real-time PCR experiments for each gene were performed on three separate occasions.

### Whole-cell patch-clamp recording

The recording and analysis of Na_v_1.8 current in rat DRG neurons was performed as previously described [16]. After 14–20 h dissociation and plating, sodium currents were recorded from the single, small-to-medium-sized DRG neurons in standard whole-cell patch-clamp configuration. All experiments were performed at room temperature (22–24°C). The pipette solution contained the following (in mM): 140 CsCl, 10 EGTA, 10 NaCl, 2 Na_2_ ATP and 10 HEPES (pH 7.3; pH adjusted with CsOH). The external solution contained the following (in mM): 35 NaCl, 65 NMDG-Cl (*N*-methyl-D-glucamine chloride), 30 TEA-Cl (tetraethylammonium chloride), 3 KCl, 5 MgCl_2_, 0.1 CaCl_2_, 0.1 CoCl_2_, 10 glucose, and 10 HEPES (pH 7.4; pH adjusted with NaOH). DRG neurons were pretreated with CCL2 (10 nM), CCR2 inhibitor INCB3344 (10 nM) and/or PKC inhibitor Sotrastaurin (AEB071; 5 μM) (Sigma-Aldrich) overnight.

To isolate TTX-R currents, 30 mM TEA-Cl, 0.1 mM CoCl_2_, and 300 nM TTX were included in the external solution to inhibit endogenous K^+^, Ca^2+^ and TTX-S Na^+^ currents. The pipette solution was used in these studies to facilitate the separation of the slowly inactivating Na_v_1.8 TTX-R current from the persistent Na_v_1.9 TTX-R current.

The membrane current or voltage was recorded using an Axopatch-200 B amplifier and Clampex 9.2 software (Molecular Devices), low-pass filtered at 2 kHz, and sampled at 50 kHz for DRG neurons. The pipette potential was zeroed before seal formation and note that the amplitude of Na^+^ currents was not affected over the time period. Five minutes after establishing the whole-cell configuration, currents were recorded every 2 min until the maximal peak current was measured. To analyse the voltage dependence of activation, currents were evoked by 10-ms pulses ranging from −70 to +40 mV in steps of 10 mV and this protocol was started at 10 min after the recording was established.

The peak current values at each potential were plotted to generate I–V curves. G values were determined by the following equation: G=I/(V_m_−V_rev_), where I is the current, V_m_ is the potential at which the current is evoked, and V_rev_ is the reversal potential. Activation curves were fitted with the following Boltzmann distribution equation: G/G_max_=1/{1+ exp [(V_1/2_−V_m_)/k]}, where G is the voltage-dependent sodium conductance, G_max_ is the calculated maximal sodium conductance, V_1/2_ is the potential at which activation is half-maximal, V_m_ is the test pulse voltage potential at which the current is evoked, and k is the slope. The resulting curves were normalized and fitted using a Boltzmann distribution equation as follows: I/I_max_=1/{1+exp [(V_1/2_−V_m_)/k]}, where I_max_ is the peak current elicited after the most hyperpolarized prepulse, and V_m_ is the preconditioning pulse potential.

### Immunoprecipitation

The primary DRG neurons were pretreated without CCL2 (10 nM) for 12 h and immunoprecipitation of Na_v_1.8 channels from DRG neurons was performed as previously described [17]. In brief, equal amounts of the neurons were lysed and precleared cellular lysates were incubated with A-agarose. The cleared DRG lysate was incubated with 2 μg of Na_v_1.8-specific polyclonal antibody for 2–3 h at 4°C before addition of protein A-agarose for an additional 1.5 h at 4°C. The beads were washed extensively in binding buffer and incubated with activated PKC as described below.

### *In vitro* PKC kinase assays and autoradiography

The kinase assays were performed in 200-μl reactions with the IP samples described above [25 mM HEPES, pH 7.4, 2 mM DTT (dithiothreitol), 10 mM MgCl_2_, 100 μM ATP and 5 μCi of [γ-^32^P]ATP]. Activated PKC (final concentration 1 ng/μl) (Invitrogen) was added to each reaction tube (30°C) and allowed to proceed for 2–3 min. The kinase reactions were performed in the presence or absence of the active PKC-specific inhibitor (AEB071), and were terminated with 5 vol (1 ml) of PBS containing 20 mM EDTA. Protein A beads were again collected by centrifugation and washed three times in PBS plus EDTA to further reduce the background. Proteins were eluted from the beads using the 2-lithium dodecyl sulfate–PAGE sample buffer for gel analysis. The presence of the sodium channel was verified by Western analysis using the pan-sodium channel antibody, and the phosphorylated channels were detected via autoradiography. The IP proteins were separated on NUPAGE Bis–Tris 4-12% gels (Invitrogen) using a 3-(N-morpholino)propanesulfonic acid-based buffer system. Gels for autoradiography were stained by Coomassie Brilliant Blue and then destained to compare loading levels tested in the kinase assays.

### Statistical analysis

Data are expressed as mean±S.D. All statistical analyses were carried out using SPSS 13.0 (SPSS). Statistical comparisons were performed by one-way analysis of variance followed by the Student–Newman–Keuls test. *P*<0.05 was considered statistically significant.

## RESULTS

### CCL2 up-regulates expression of CCR2 and Na_v_1.8 in DRG neurons

**Figure 1:**
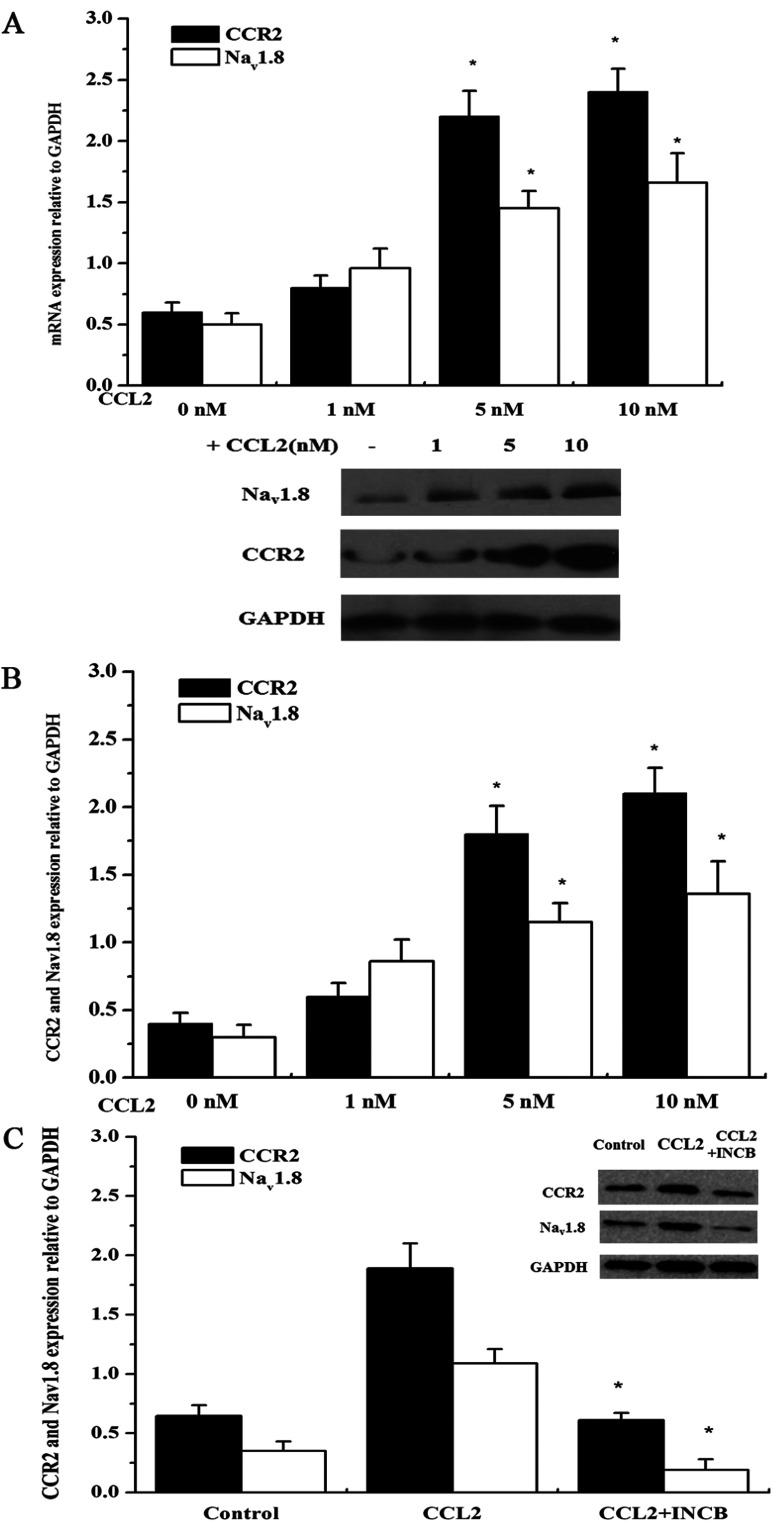
CCL2 promotes the expression of CCR2 and Na_v_1.8 in DRG neurons (A) RT–PCR analysis of the CCR2 and Na_v_1.8 mRNA levels in primary DRG neurons in response to various concentration of CCL2. (B) Western blotting analysis of CCR2 and Na_v_1.8 expression in primary DRG neurons in the absence or presence of 1, 5, 10 nM CCL2, and each band densitometry intensity was adjusted to that of the matched GAPDH control. (C) Western blotting analysis of CCR2 and Na_v_1.8 expression in response to CCR2 inhibitor INCB (10 nM) treatment. Data are means±S.D. of three independent experiments.* *P*<0.05 versus the control.

To investigate the effects of CCL2 on CCR2 and Na_v_1.8 in DRG neurons, the expression of CCR2 and Na_v_1.8 were evaluated using RT–PCR and Western blotting after the neurons were incubated with 1, 5 and 10 nM exogenous CCL2 for 24 h. As shown in Figures 1A and 1B, the expression of CCR2 and Na_v_1.8 (mRNA and protein level) increased in a dose-dependent fashion following the treatment with various concentrations of CCL2. In comparison with the control, 5 and 10 nM CCL2 significantly enhanced the expression of CCR2 and Na_v_1.8 (*P*<0.05). Thus, 10 nM CCL2 was applied for the following studies. Moreover, as shown in Figure 1(C), the administration of CCR2 inhibitor INCB3344 (10 nM) significantly blocked the CCL2-induced up-regulation of Na_v_1.8 and CCR2 compared with the sole CCL2 treatment (*P*<0.05).

### CCL2–CCR2 are involved in the regulation of Na_v_1.8 currents in DRG neurons

**Figure 2:**
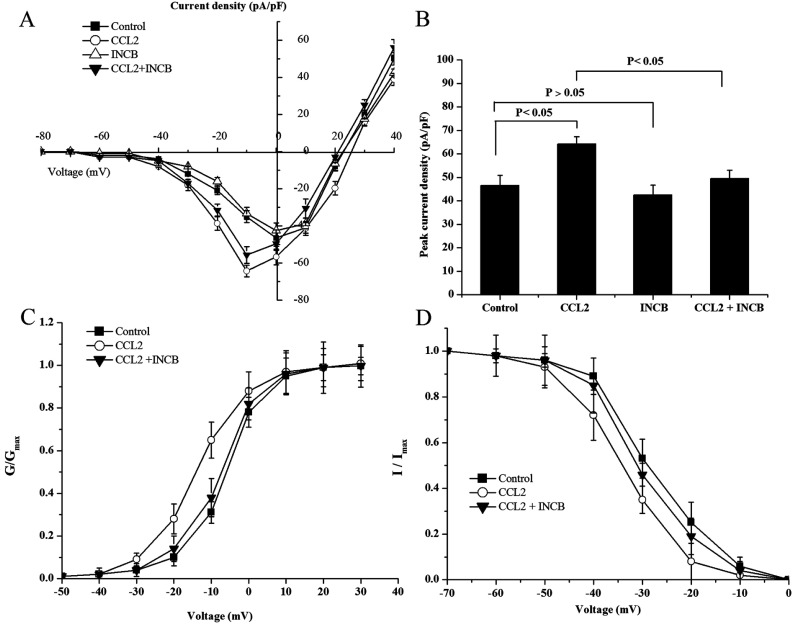
CCR2 inhibitor blocks the elevated Na_v_1.8 current induced by CCL2 in DRG neurons (A) Current–voltage relationships of Na_v_1.8 were determined before and after treatment with CCL2 (10 nM) or CCR2 inhibitor INCB (10 nM), alone or in combination. (B) Peak current density of Na_v_1.8 in response to different treatments. The activation (C) and inactivation (D) curve of Na_v_1.8 current in the presence of CCL2(10 nM) or CCL2 and INCB (10 nM). Data are means±S.D. of three independent experiments.

Using a whole-cell patch-clamp configuration, we evaluated the effects of CCL2–CCR2 on the density and kinetic properties of Na_v_1.8 sodium currents in DRG neurons. In our study, the family of Na_v_1.8 sodium currents was generated by applying a range of membrane potentials between -70 mV and +40 mV for 100 ms according to the previous reports. The current–voltage (I–V) analysis revealed that the mean peak current amplitude was significantly higher in CCL2-treated neurons than in control neurons (64.28±3.12 pA/pF versus 46.53±4.33 pA/pF, *P*<0.05). However, the mean peak current amplitude of Na_v_1.8 in DRG neurons was significantly reduced after being treated with CCL2+CCR2 inhibitor INCB3344 (10 nM) compared with the control and CCL2-treated neurons (*P*<0.05), whereas no significant change in Na_v_1.8 current density was observed when INCB3344 (10 nM) was applied alone (Figures 2A and 2B). Furthermore, we found that the application of CCL2 shifted the activation and steady-state inactivation curves of Na_v_1.8 in a hyperpolarizing direction, and this effect was prevented and returned to baseline levels by INCB3344 (Figures 2C and 2D).

### PKC is associated with CCL2-induced Na_v_1.8 currents by promoting the phosphorylation of Na_v_1.8

**Figure 3:**
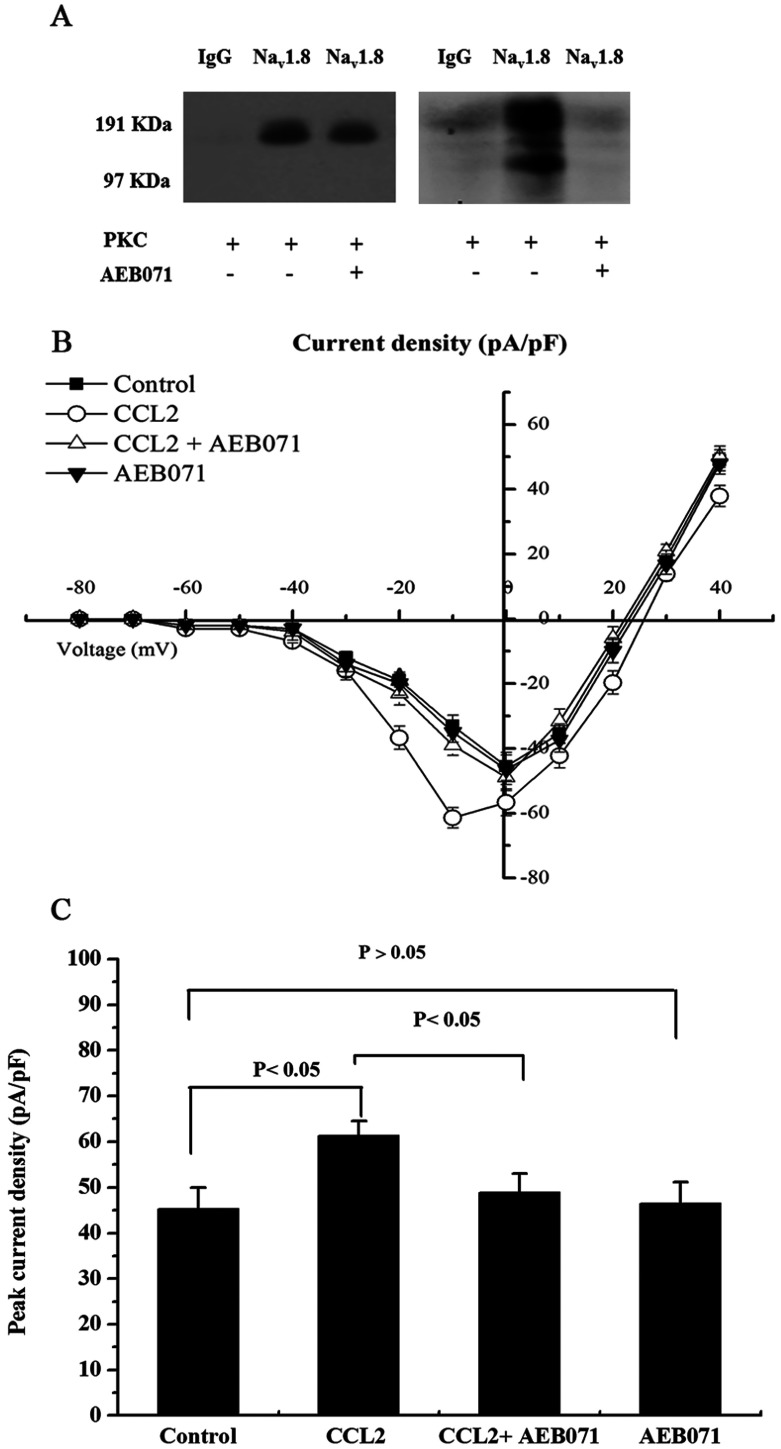
Na_v_1.8 is phosphorylated by activated PKC (A) Lane 1 of the Western blot (left panel) and the autoradiography (right panel) show that neither protein product was immunoprecipitated nor phosphorylated by the control IgG antibody. Lanes 2 and 3 of the Western blot demonstrate that equal levels of immunoprecipitated Na_v_1.8 channel protein were used in the kinase assay. Lanes 2 and 3 of the autoradiography demonstrate that PKC phosphorylation of the Na_v_1.8 immunoprecipitated product is only observed in the absence of PKC inhibitor (AEB071). (B) Current–voltage relationships of Na_v_1.8 were determined before and after the treatment with CCL2 (10 nM) or PKC inhibitor AEB071, alone or in combination. (C) Peak current density of Na_v_1.8 in response to different treatment.

In order to investigate the role of PKC in CCL2-induced inflammatory nociception, the effects of PKC on the peak current density of Na_v_1.8 were measured using whole-cell patch-clamp protocols. It was showed that after inhibition of PKC by AEB071, the CCL2-induced increase of peak current density of Na_v_1.8 was significantly reversed (*P*<0.05) while no significant decrease was observed when AEB071 was applied alone compared with the control (Figures 3A and 3B).

To further elucidate whether PKC was responsible for the phosphorylation of Na_v_1.8, *in vitro* PKC kinase assays were performed to detect the phosphorylated Na_v_1.8. After the DRG cells were pretreated with CCL2 for 30 min, the native Nav1.8 channels were immunoprecipitated using a Na_v_1.8 antibody and then were incubated with the activated recombinant PKC and [γ-^32^P]ATP in the presence or absence of AEB071. A control IgG antibody was used to validate the specificity of the IP reaction and kinase assay. The Western blot assay indicated that the Na_v_1.8 antibody used in the IP reaction had a high specificity. The autoradiogram demonstrated that Na_v_1.8 is a substrate of PKC and that the inhibition of PKC eliminated the generation of phosphorylated Na_v_1.8 (Figure 3C).

### PKC activates NF-κB to promote Na_v_1.8 expression

**Figure 4:**
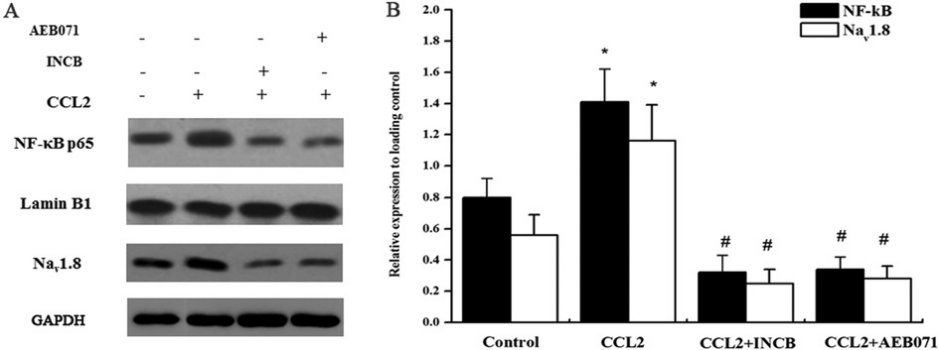
PKC activates NF-κB to promote Na_v_1.8 expression (A) Western blotting analysis of expression of Na_v_1.8 and nuclear NF-κB p65 subunit in primary DRG neurons in the absence or presence of CCL2, INCB and AEB071. (B) Each band densitometry intensity was adjusted to that of the matched loading control. GAPDH and Lamin B1 are set as the cytoplasm and nuclear protein controls, respectively. Data are means±S.D. of three independent experiments.*P<0.05 versus the control. #*P*<0.05 versus the CCL2 treatment.

It has been demonstrated that PKC is responsible for the activation of NF-κB, and in turn, we determined whether the PKC–NF-κB pathway is implicated in the modulation of Na_v_1.8 expression since we observed that Na_v_1.8 was unregulated in response to the treatment of CCL2. Western blot assays were performed to analyse the expression of p65 and Na_v_1.8 when treated with CCL2 or PKC inhibitor. As shown in Figures 4(A) and 4(B), compared with the control, CCL2 (10 nM) significantly induced the nuclear p65 expression and the up-regulation was reversed after PKC inhibition with AEB071, and correspondingly the expression of Na_v_1.8 changed in response to the p65 expression.

## DISCUSSION

Although multiple lines of evidence indicate that CCL2 participates in the pathogenesis of neuropathic pain, the mechanisms underlying the pain regulation are poorly understood. In the present study, we showed that CCL2 induced an increase in the VGSC Na_v_1.8 current density and expression, and that PKC–NF-κB were involved in the regulation of this phenomenon.

Pain is a sensory mechanism that warns us of imminent tissue damage. Nociceptors located at the terminals of primary afferent neurons transduce noxious chemical, mechanical or thermal stimuli into depolarizing currents that ultimately induce action potentials [18]. Emerging studies have demonstrated a predominant role of inflammatory chemokines and cytokines in pain, which can directly activate or modify the activity of nociceptors, contributing to pain hypersensitivity and spontaneous pain [19]. Among them, CCL2 has an important role in modulation of nociception, and accordingly, CCR2 has been proven to be expressed in the DRG and spinal cord in neurons [20]. Moreover, the administration of CCL2 to the experimental animal evokes hypernociceptive behavioural reactions [21]. Kiguchi et al. [2] reported that epigenetic up-regulation of CCL2 was associated with the modulation of CCR2. In accordance with the previous studies, we also observed the elevated expression of CCR2 in DRG neurons in response to the treatment with CCL2 *in vitro*.

As an important member of the VGSCs that are responsible for nerve impulse conduction, Na_v_1.8 was considered to mostly contribute to the enhanced excitability and spontaneous ectopic discharges occurring in small- and medium-sized DRG neurons [7]. Although it was confirmed that Na_v_1.8 was both expressed on small and medium diameter L4 DRG neurons in different proportions (83.2% of all small diameter cells <700 μm^2^, 17.2% of all medium-to-large diameter cells >700 μm^2^, n=3), it is still a limitation that we failed to isolate the small-sized DRG neurons for the present study. Kao et al. [3] reported that CCL2 up-regulated the current density and expression of TRPV1 (transient receptor potential vanilloid 1) and Na_v_1.8 in DRG neurons. Belkouch et al. [22] revealed that CCL2 concentration dependently increased TTX-R Na_v_1.8 current densities in both small- and medium-diameter sensory neurons, which was reversed by the administration of CCR2 antagonist INCB. Furthermore, Gβγ was suggested to be involved in the crosstalk between CCL2 and Na_v_1.8. Our study partly confirmed the previous conclusions that the CCL2–CCR2 axis participate in the modulation of Na_v_1.8 expression and current density. Meanwhile, we found that PKC–NF-κB contributed to the regulation of Na_v_1.8 expression and current density, another underlying mechanism that was implicated in Na_v_1.8 modulation.

It is believed that Na^+^-channel function is affected by phosphorylation [23] and PKC exerts an function in the phosphorylation of Na^+^ channels. It was also found that all α-subunits of VGSCs share a common topology and contain several potential PKC phosphorylation sites in the first and third intracellular loops. Chen et al. [24] proved that PKC phosphorylates brain Na^+^ channels and reduces peak Na^+^ current in hippocampal neurons. Chang et al. [25] found that neurokinin-1 receptor activation potentiated Na_v_1.8 current in a PKC-dependent pattern. Moreover, a compelling study determined that Na_v_1.8 was a substrate of PKC that mediates mechanical hyperalgesia [16]. Therefore we investigated the role of PKC in the CCL2-induced pathogenesis of pain.

In the present study, we found that the inhibition of PKC by AEB071 significantly reduced the CCL2-induced Na_v_1.8 peak current densities, and the *in vitro* PKC kinase assays also confirmed that PKC contributed to the phosphorylation of Na_v_1.8 in response to CCL2. Furthermore, regarding the CCL2-induced up-regulation of Na_v_1.8, we evaluated the role of the PKC–NF-κB pathway in this phenomenon, and found that CCL2 induced the activation of NF-κB, whereas the inhibition of PKC and NF-κB reduced the Na_v_1.8 expression. However, more details about the effects of CCL2 on PKC as well as the interaction of PKC and Na_v_1.8 await clarification.

In summary, our findings demonstrate that CCL2 induced the up-regulation and an increase of peak current density of Na_v_1.8 in DRG neurons. PKC contributed to the CCL2-induced inflammatory hyperalgesia through promoting the phosphorylation of Na_v_1.8 and activating NF-κB to modulate the expression of Na_v_1.8.
